# Detection of BCR/ABL Translocation in Bone Marrow Derived Mesenchymal Stem Cells in Egyptian CML Patients

**DOI:** 10.3889/oamjms.2015.040

**Published:** 2015-04-08

**Authors:** Taghrid Mohamed Gaafar, Inas Ismail Raafat, Azza Ahmed Aly, Nagwa Abd EL-Ghaffar Mohamed, Reem Jan Farid, Neveen Ezzat Saad, Rabab EL-Hawary, Naglaa Mostafaa, Mirhan Mohamed Ahmed

**Affiliations:** 1 *Department of Clinical and Chemical Pathology, Faculty of Medicine, Cairo University, Cairo, Egypt*; 2 *Department of Clinical and Chemical Pathology, National Research Center, Egypt*; 3 *Department of Clinical and Chemical Pathology, National Cancer Institute, Cairo, Egypt*

**Keywords:** Chronic myeloid leukemia, mesenchymal stem cell, Philadelphia chromosome

## Abstract

**BACKGROUND:**

Chronic myeloid leukemia (CML) is a clonal myeloproliferative disorder of hematopoietic stem cells. It is characterized at the cytogenetic level by Philadelphia (ph) chromosome and at the molecular level by the BCR/ABL gene rearrangement. Bone marrow derived mesenchymal stem cells (MSCs) are pluripotent stem cells that can differentiate into several mesenchymal tissues.

**AIM:**

To observe the biological characteristics of MSCS from CML patients and to determine whether MSCs harbor the abnormal BCR/ABL translocation similar to CML bone marrow cells.

**SUBJECTS AND METHODS:**

Characterized MSCs were isolated from 12 newly diagnosed Philadelphia positive untreated CML patients.

**RESULTS:**

MSCs can be readily isolated from CML marrow and exhibit major expansion. Flow cytometry analysis revealed the typical MSC phenotype. Moreover; MSCs do not harbor the BCR/ABL translocation confirmed by karyotype and real time PCR.

**CONCLUSION:**

MSCs from CML patients express the typical MSC phenotype; and do not express the BCR/ABL gene. Since; MSCs are able to support engraftment of hematopoietic stem cells in stem cell transplantation(SCT) as well as suppress alloreactive T cells causing graft versus –host disease, this current study provides evidence that in a SCT setting of CML patients, autologous MSCs could be a source of stem cell support in future cell therapy applications.

## Introduction

Bone marrow (BM) is composed of at least two systems, the hematopoietic tissue proper and the stroma. Mesenchymal stem cells (MSCs) are important constituents of this microenvironment and are characterized as adult, non-hematopoietic stem cells (HSCs) which, after an adequate stimulus, can differentiate morphologically and functionally into different cell lines including the stroma, which gives support to hematopoiesis, adipocytes, chondrocytes, myocytes, astrocytes, tenocytes, and hepatocytes [1].

Chronic myeloid leukemia is a clonal myeloproliferative disorder of hematopoietic stem cells. It is characterized at the molecular level by BCR/ABL gene re-arrangement [2]. This rearrangement results in a shortened chromosome 22, designated the Philadelphia (Ph) chromosome [3].

Previous studies have suggested that CML stem cells might be more primitive stem cells than multipotent HSCs when the BCR/ABL fusion gene in haemangioblastic-like cells have been identified [4] while other studies found the BCR/ABL fusion gene in even more primitive progenitor cells defined as Flk1^+^, CD31^-^, CD34^-^ [5].

All these findings raised the question whether the BCR/ABL fusion gene can also be found in MSCs in patients with CML. Since Ph+biphenotypic leukemia may arise from even more immature stem cells than CML, the potential relation of such stem cells to MSCs would also be of interest. In fact, whereas some recent data suggest that MSCs in chronic phase CML may not display clonal markers of CML, the more primitive variants of Ph+ neoplasms have not been investigated for such a relationship so far [6].

CML marrow is an abundant source of MSCs not to be involved by the malignant process of CML. Furthermore, these MSCs from a CML patient could support in vitro cord blood expansion as those MSCs from a normal donor [2].

Allogeneic stem cell transplantation (allo-SCT) is the only curative treatment for CML as well as many hematological malignancies and other immunohematopoietic disorders [7].

According to recent studies [8] MSCs do not express the BCR/ABL gene so compare to allogeneic MSCs, autologous MSCs from patients needed cell- based therapy may be an ideal alternative stem cell source. However, characteristics of MSCs from a disease state are poorly understood [9].

The aim of this work is isolation and characterization of MSCs from CML patients and demonstration of whether MSCs from CML patients harbor the abnormal Ph chromosome, since autologous Philadelphia negative MSCs could be a source of stem cell support in future cell therapy applications.

## Subjects and Methods

This study has been approved by the ethical comity of Cairo University and National Research Center. The study involved fifteen patients admitted to National Institute of Cancer with newly diagnosed Ph+ve CML as verified by karyotyping and FISH of their bone marrow aspirate. After informed consent had been obtained from all patients, 2 ml heparinized bone marrow samples were obtained by bone marrow aspirate from iliac crest and were used for:

### Isolation and culture of BM derived MSC

BM mononuclear cells were separated by density gradient centrifugation using Biocoll separating solution; density 1.077 g/ml (Biochrom AG). Briefly, 5 ml of heparinized BM cells were mixed with equal volume of Dulbecco’s modified Eagle’s Medium (DMEM) (Hyclone, Thermscientific) and layered slowly over the Biocoll then centrifuged at 400xg for 20 min at room temperature.

The mononuclear cells were collected from the interface and washed twice with DMEM containing 10% fetal calf serum (FCS, Euroclone). Total cell count and viability were evaluated by 0.2% Trypan blue exclusion.

A total of 1x10^6 cells/ml of BM mononuclear cells were cultured in DMEM complete medium supplemented with 10% fetal calf serum (FCS, Euroclon) to promote their optimal expansion, 1% antibiotic/antimicotic (Hyclone) was added to prevent bacterial and fungal infection. Cultures were kept then in suitable environmental conditions e.g. (37°C in humidified 5% CO_2_ air incubator).

On day 3 of culture, non-adherent cells were discarded and fresh medium was added. Media was changed twice/week. When adherent MSCs reach 80-90% confluent, they were trypsinized by 0.25% trypsin EDTE (Hyclon), 5 min at 37°C then washed twice with complete media and passaged for the next expansion [10].

### Flow cytometry analysis of cultured BM derived MSCs

Immunophenotyping of BM derived MSCs was done as follow [8]. At the end of the third passage, cells were trypisinized and adjusted to 1x10^6 cells/ml. then 1x10^6 cells were incubated with 10 µL of monoclonal antibodies as determined from the manufacturer’s recommendation: CD 105 PE (mesenchymal marker), and exclusion markers CD34 FITC (hematopoietic stem cell marker) at 4°c in the dark. As a control unstained cells were applied first to exclude the effect of autofluoresence of the cultured cells. After 20 min incubation, 2ml of PBS containing 2% FCS solution were added to each tube of monoclonal treated cells. The mixtures were then centrifuged for 5 min at 2500 rpm followed by discarding the supernatant and resuspending cells in 500µl PBS containing 2%FCS. Finally, cell immunophenotyping was performed using CYTOMICS FC 500 flow cytometer (Beckman coulter, FL, USA) and analyzed using CXP software version 2.2.

### Karyotypic analysis of hMSCs

Trypsinized MSC were washed twice with Hank’s balanced salt solution (Lonza). One to two millions of cells were cultured in 20% fetal bovine serum/RPMI -1640 (Hyclone) in CO_2_ incubator overnight. Cells were fixed with a mixture of glacial acetic acid and absolute methanol (1:3 by volume). Finally, metaphase cells were dropped onto microscope glass slides. Giemsa-Trypsin (GTG) banded chromosomes were prepared and analyzed.

### Reverse transcriptase polymerase chain reaction (REALTIME PCR)

Total cellular RNA was extracted from cultured MSCs using high pure RNA isolation kit (Roche cat NO.11 828 665 001), after cDNA synthesis (Roche transcriptor first strand cDNA synthesis kit cat.NO. 04 896 866 001), amplification of the fusion gene BCR-ABL & hybridization were performed using light cycler 2& Roche light cycler fast DNA master HybProbe (cat no.03 003 248 001). Amplification of the ABL gene as internal control was done for each sample. Expression of target gene measured relative to mean critical CT values of the house keeping gene.

## Results

The present work included fifteen patients (n=15) with newly diagnosed Ph+ve CML as verified by karyotyping and FISH. Of the fifteen patients, nine (60%) were males and six (40%) were females. The age ranged between 34-55 years with a mean of 44.4 ± 6.8 years. TLC ranged from (180 000-320 000/mm^3^) with mean of 233.333 ± 45. 3. Individual and clinical data of patients are shown in Table 1.

**Table 1:** Individual and clinical data of CML patients.

Patient	Sex	Age	TLC/mm^3^
1	male	40	180,000
2	female	43	210,000
3	female	34	290,000
4	male	35	190,000
5	male	40	300,000
6	female	43	230,000
7	male	49	250.000
8	male	50	180.000
9	male	51	260,000
10	male	50	320.000
11	male	37	250.000
12	female	39	180,000
13	male	55	200,000
14	female	48	240,000
15	female	53	220,000

A cytogenetic study by karyotyping and FISH analysis of bone marrow cells revealed the presence of the Ph chromosome in all patients.

**Table 2:** Cytogenetic and molecular analysis of BM and BM- derived MSCs of CML patients.

Patient	CML stage	Bone marrow cells		Bone marrow derived MSCs	

		Karyotype	FISH	Karyotyping	RT - PCR
1	Chronic	46xy 30%, 46xy,t(9,22)(q34;q11) 70%	Positive	………..	………..
2	Chronic	46xx,t(9,22)(q34; q11) 100%	Positive	…………	…………
3	Chronic	46xx,t(9,22)(q34; q11) 100%	Positive	………….	…………
4	Chronic	46xy,t(9,22)(q34; q11) 100%	Positive	Negative	Negative
5	Chronic	46xy,t(9,22)(q34; q11) 100%	Positive	Negative	Negative
6	Chronic	46xx,t(9,22)(q34; q11) 100%	Positive	Negative	Negative
7	Chronic	46xy,t(9,22)(q34; q11) 100%	Positive	Negative	Negative
8	Chronic	46xy,t(9,22)(q34; q11) 100%	Positive	Negative	Negative
9	Chronic	46xy,t(9, 14,22)(q34; q22; q11)100%	Positive	Negative	Negative
10	Chronic	46 xy 20% 46xy,t(9,22)(q34 q11) 80%	Positive	Negative	Negative
11	Chronic	46xy,t(9,22)(q34 q11) 100%	Positive	Negative	Negative
12	Chronic	46xx,t(9,22)(q34 q11) 100%	Positive	Negative	Negative
13	Chronic	46xy,t(9,22)(q34; q11) 100%	Positive	Negative	Negative
14	Chronic	46xx,t(9,22)(q34 q11) 100%	Positive	Negative	Negative
15	Chronic	46xx,t(9,22)(q34 q11) 100%	Positive	Negative	Negative

### Isolation and expansion of BM derived MSCs

**Figure 1:**
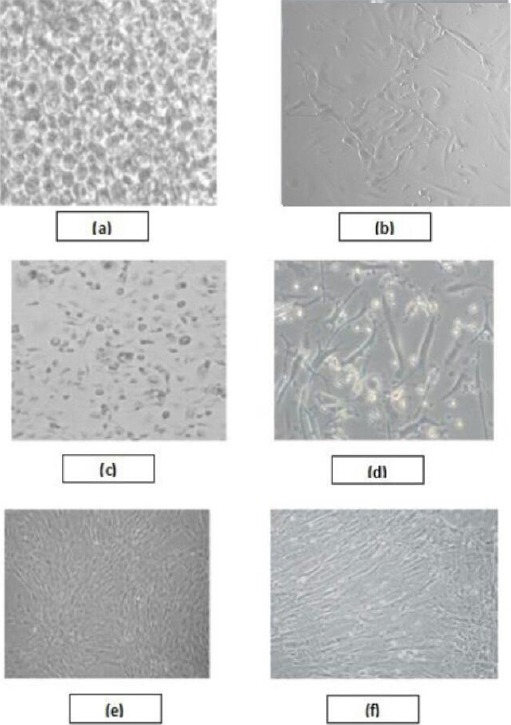
*Bone Marrow derived MSCs under inverted microscope at different days of culture. (a) Day 0: mononuclear cells isolated from BM-MSCs, (b) Day 5: MSCs colony forming unit, (c) Day 10 MSCs with some hematopoietic cells contamination, (d) Day 18:spindle shaped MSCs with few contaminating hematopoietic cells, (e) Day 21 and (f) Day 21 confluent growth 80-90%)*.

From the 15 BM samples collected: Two samples (13.33%) were discarded due to contamination (sample 1 and 2). One sample (6.66%) showed few adherent spindle shaped cells, however, these cells failed to grow and expand in the culture conditions (sample 3). Spindle like cells were expanded from 12 samples (80%), where confluence was reached after 3 weeks. Cells were then splitted using Trypsin EDTA and replated at a density of 5000 cells/cmm and were maintained till the third passage. MSCs are characterized by: 1) Plastic adherence that resist washing while changing the media and 2) Spindle fibroblast like morphology under the inverted microscope. Images were captured with a digital camera as shown in Figure 1.

### Immunophenotyping

**Figure 2:**
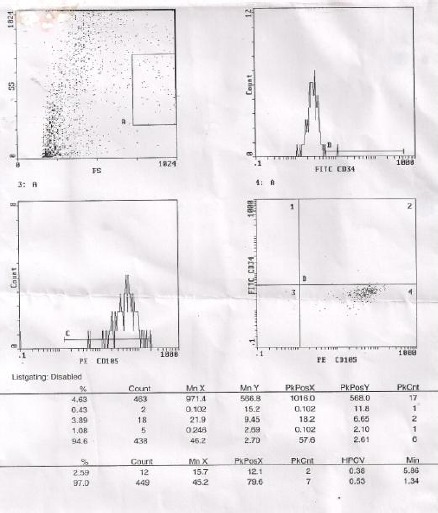
*The immunophenotypic analysis of cultured hMSCs isolated from the bone marrow of CML patients*.

The cells were positive for CD 105 (97%). No detectable contamination of hematopoietic cells was observed, as flow cytometry analysis was negative for markers of hematopoietic lineage CD34 (2.59%), as shown in Figure 2.

### Ph chromosome assessment

#### a) Karyotypic analysis of MSCs:

**Figure 3:**
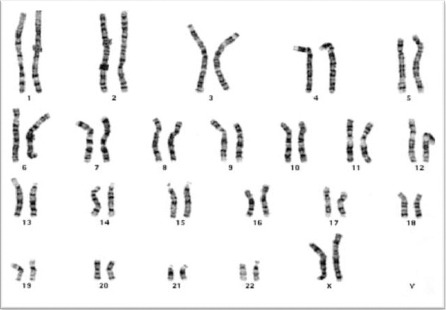
*The karyotype of cultured hMSCs isolated from the bone marrow of female CML patients show normal pattern*.

**Figure 4:**
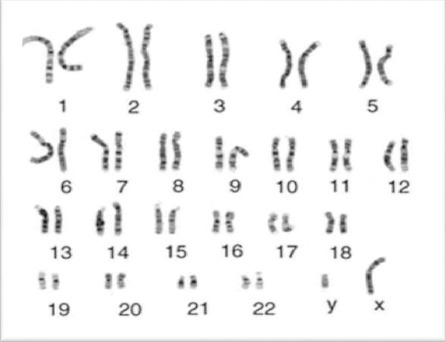
*The karyotype of cultured hMSCs isolated from the bone marrow of male CML patients show normal pattern*.

Cultured MSCS showed no cytogenetic aberrations when analyzed with conventional chromosomal staining (Fig. 3, 4).

### Real Time Polymerase Chain Reaction

Interpretation of real time PCR curves (Table 3):

**Table 3:** Analysis of results (Light Cycler^®^ 2.0 Instrument, Roche Master: Fast Start).

BCR-ABL t(9;22) (sample)	NTC	Result
No amplification	Negative	Negative
Amplification signal	Negative	Positive for BCR-ABL t(9;22) fusion transcripts
Amplification signal	Positive	Contamination, repeat experiment

In all of the twelve BM derived MSCs samples, negative control (NTC) showed no signal.

**figure 5:**
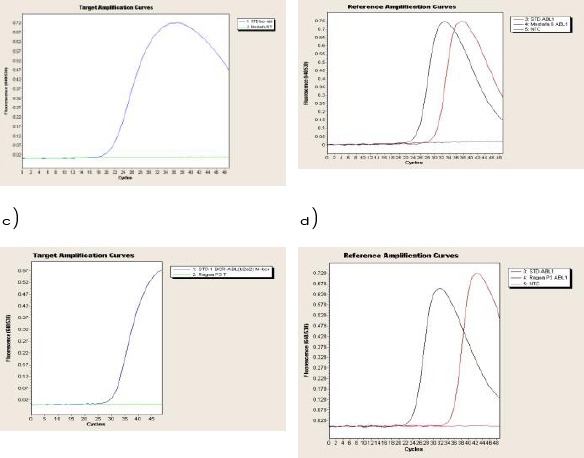
*a) Target amplification curve of Standard BCR-ABL (blue line) compared to that of a male patient (green line). A flat green line indicating no BCR-ABL translocation compared with that of standard. b) Reference amplification curve of Standard ABL1 (red line) compared to the housekeeping gene ABL1 (grey line) of a male patient. Both crossing the negative control (blue line), indicating successfully amplified patient RNA. c) Target amplification curve of Standard BCR-ABL (blue line) compared to that of a female patient (green line). A flat green line indicating no BCR-ABL translocation compared with that of standard. d) Reference amplification curve of Standard ABL1 (red line) compared to the housekeeping gene ABL1 (grey line) of a female patient. Both crossing the negative control (pink line), indicating successfully amplified patient RNA*.

− The provided standard row of cloned and purified DNA with concentrations in the range from 10^6^ copies/rxn to 10^1^ copies/rxn of both housekeeping gene (ABL1) and BCR-ABL t(9;22) fusion transcript DNA b2a2 had CPs between cycles 18 and 35 (as described by manufacturer’s instructions).

− The BCR-ABL t(9;22) fusion transcripts was not detected as shown in Figure 5.

## Discussion

Chronic myeloid leukemia (CML) is a hematopoietic stem cells cancer driven by the BCR-ABL fusion protein that arises from translocation of chromosomes 9 and 22. Cytoreductive chemotherapy, such as busulfan and hydroxyurea was a mainstay of therapy to control WBC counts, however, it did not modify the progression of the disease to accelerated phase and blastic phase. Allogeneic BMT is the only known curative therapy for CML; however, treatment-related mortality from infection, bleeding, and graft versus host disease, age, and availability of suitable donors limits the widespread use [11].

Mesenchymal stem cells are multipotent, which means that they are able to differentiate into several lineages. Together with hematopoietic stem cells and endothelial stem cells, the MSCs form the progenitor cells in the bone marrow. They can stimulate and differentiate the blood cells in the bone marrow due to production of growth factors and cytokines. MSCs are generally known from their target in tissue regeneration fields, however, the role of the MSCs in the development and progression of leukemia is still unknown [12]. MSCs have been found to suppress inflammation by inhibiting T cell proliferation, representing a novel treatment for graft –versus host disease (GVHD). In addition, MSCs seem not to suppress the whole immune system but specifically a GVHD without impairment of the graft versus leukemia effect in leukemia patients [13].

In the present work we succeeded to isolate and expand MSCs from 80% of CML patients. The high homogeneity of MSCs in present study was consistent with [14, 15].

In the current study, adherent MSCs derived from bone marrow of CML patients gave rise to colonies and exhibited characteristic spindle-shaped morphology after 7 days and they reached 90% confluency after an average of 21 days. This was in line with study that described their CML derived MSCs by showing dense growth after a median of 7 days [6]. Then, cells started to grow out of highly proliferative centres and spread radiantly throughout the culture. These centres were only seen before the first passage and disappeared afterwards.

In the present study immunophenotyping analysis of MSCs by flow cytometry show that MSCs were positive for CD105 and negative for the hematopoietic marker CD45. This was in accordance with [6, 8] who described isolated MSCs from CML patients to be highly positive for CD73, CD90 and CD105 and negative for CD34 and CD45.

In our study, karyotyping analysis of MSCs showed absence of Philadelphia chromosome. We have also investigated the presence of BCR-ABL translocation in CML patients derived MSCs by Real time PCR. Real time PCR demonstrated that the BCR/ABL fusion gene was not present in bone marrow derived MSCs of CML patients despite its presence in the corresponding bone marrow cells. These findings were in agreement with [9, 12] who demonstrated that CML derived MSCs did not express BCR/ABL gene and Philadelphia chromosome and had not the ability to develop tumor in nude mice

In contrast to our results [8] had characterized phenotypically and cytogenetically a population of MSCs isolated from BM of 5 Ph+ve untreated CML patients. They analyzed the BCR-ABL gene by fish in 1513 nuclei. They only detected 0.01% (2/1513) BCR-ABL positive MSCs. They referred the detection of these two BCR-ABL positive nuclei to the presence of hematopoietic contamination in the culture of these cells, possibly (CD14) BCR-ABL positive macrophages that grow adhering to MSC.

Also it was in accordance with our results as their results suggested that cultured MSCs in patients with BCR-ABL positive CML do not harbor this specific translocation [6]. Apart from two samples that were positive for BCR-ABL when analyzed with a highly sensitive RT-PCR method typically used for detecting minimal residual disease CML patients. They suggested a contamination of the MSC population with hematopoietic cells, most likely macrophages as Fluorescence Activated Cell Sorting (FACS) analysis of these samples showed a small CD45 positive cell population

Being in line with authors who described BCR-ABL positive macrophages contaminating in vitro cultures of stromal cells from BM of CML patients, Wὃhrer et al., 2007 showed that the abnormal stromal function in CML might in fact be due to the presence of BCR-ABL positive macrophages in the marrow microenvironment which may contribute to selective expansion of leukemic hematopoietic cells [16].

Tyrosine kinase (TK) inhibitor drugs specifically target BCR-ABL, the constitutively activated tyrosine kinase fusion protein and revolutionized treatment of CML [17].

Bone marrow transplantation was also used as initial treatment of CML before advent of Imatinib tyrosine kinase inhibitor, but in now rarely used, primarily for those who fail drug treatment (Kimura et al., 2006). Stem cell transplantation remains an option for those patients who rarely develop T3151 mutation [18], refractory cases to chemotherapy, Philadelphia negative CML & cases resistant to inhibitors [19].

MSCs are known to interact with HSCs and immune cells, and represent potential cellular therapy to enhance allogeneic hematopoietic engraftment and prevent GVHD. Co-culture of MSCs and HSCs could cause a significantly increase in CD34+ve cells [20].

MSCs negative for the malignant transformation could be an important tool used to alleviate GFHD. Lazarus et al., reported that autologous MSCs, derived from hematologic malignancies bone marrow, could be infused intravenously in those patients without toxicity. In addition, the incidence of acute and chronic GVHD was significantly reduced in patients co-transplanted with both MSCs and HSCs [21].

However, the safety of MSCs co-transplantation should be more investigated as some studies found that the capacity of patient Flk(+), CD3 (-), CD34(-) MSCs to inhibit T lymphocyte activation and proliferation was impaired in vitro. They also found that CML patient-derived MSCs have impaired immuno-modulatory functions, suggesting that the dysregulation of hematopoiesis and immune response may originate from MSCs rather than HSCs [22].

In conclusion, the present study demonstrated that isolated MSCs from the bone marrow of patients with CML are negative for BCR/ABL translocation. So we suggest that autologous MSCs could be co-transplanted with HSCs in CML treatment. Moreover, co-transplantation of allogeneic hematopoietic stem cells and MSCs devoid of malignant transformation can facilitate the implantation of hematopoietic cells & alleviate GVHD through their regulatory immune response approach. Finally, from our findings and similar observations by [2, 6, 8], it may be suggested that autologous MSCs can be used in this setting without running the risk of re-transplantation of leukemic stem cells.
